# Brentuximab vedotin related bilateral Purtscher-like retinopathy unresponsive to pulse steroid therapy and intravitreal aflibercept injection

**DOI:** 10.3205/oc000080

**Published:** 2017-12-15

**Authors:** Ziya Ayhan, Sureyya Yigit Kaya, Mehmet Ali Ozcan, Ali Osman Saatci

**Affiliations:** 1 Department of Ophthalmology, Dokuz Eylul University School of Medicine, Izmir, Turkey; 2 Department of Haematology-Oncology, Dokuz Eylul University School of Medicine, Izmir, Turkey

**Keywords:** Brentixumab vedotin, Hodgkin’s lymphoma, Purtscher-like retinopathy

## Abstract

We describe a 36-year-old woman with a relapsing Hodgkin’s lymphoma who developed a severe bilateral sudden visual loss almost three weeks after the initiation of brentuximab therapy. Ancillary fundus tests yielded bilateral severe retinal arteriolar occlusion 360° and serous macular retinal detachment. No visual improvement could be achieved despite the pulse corticosteroid therapy and a single bilateral intravitreal aflibercept administration cessation of the brentuximab therapy. Unfortunately, she succumbed to respiratory failure almost six weeks after the diagnosis of Purtscher-like retinopathy.

## Introduction

Purtscher-like retinopathy is an occlusive microvasculopathy most often developed after cranial trauma, thoracic compression, acute pancreatitis, renal failure, various autoimmune disease and during the administration of a multitude of chemotherapeutic agents [1], [2], [3]. Brentuximab vedotin is a targeted antibody-drug conjugate active against CD-30 positive cancer cells such as those associated with classical Hodgkin’s lymphoma and it becomes an important option for the management of patients who have failed high dose chemotherapy/autologous hematopoietic stem cell transplantation (AHSCT) or at least two prior chemotherapy regimens and as post-AHSCT consolidation therapy in patients who are at increased risk of relapse or progression after AHSCT [4], [5]. We describe a patient with refractory Hodgkin’s lymphoma who developed Purtscher-like retinopathy after administration of brentuximab vedotin following HSCT. 

## Case description

A 36-year-old woman was referred to us by her haematologist for a sudden, severe, bilateral visual loss of two days’ duration. She was diagnosed to have stage 2B nodular sclerosing classic Hodgkin’s lymphoma almost a year ago and first underwent six cycles of adriablastin, bleomycin, vinblastine, dacarbazine (ABVD regimen) and then AHSCT due to progression of the disease process. However, early relapse was detected by comparing the baseline and control scans of positron emission tomography/computed tomography (PET/CT) after the AHSCT. Therefore, brentuximab vedotin (1.8 mg/kg once every 3 weeks) was administered for the consolidation therapy. However, she experienced bilateral, sudden, severe visual loss almost three weeks after the initiation of brentuximab therapy. 

**Figure 1:**
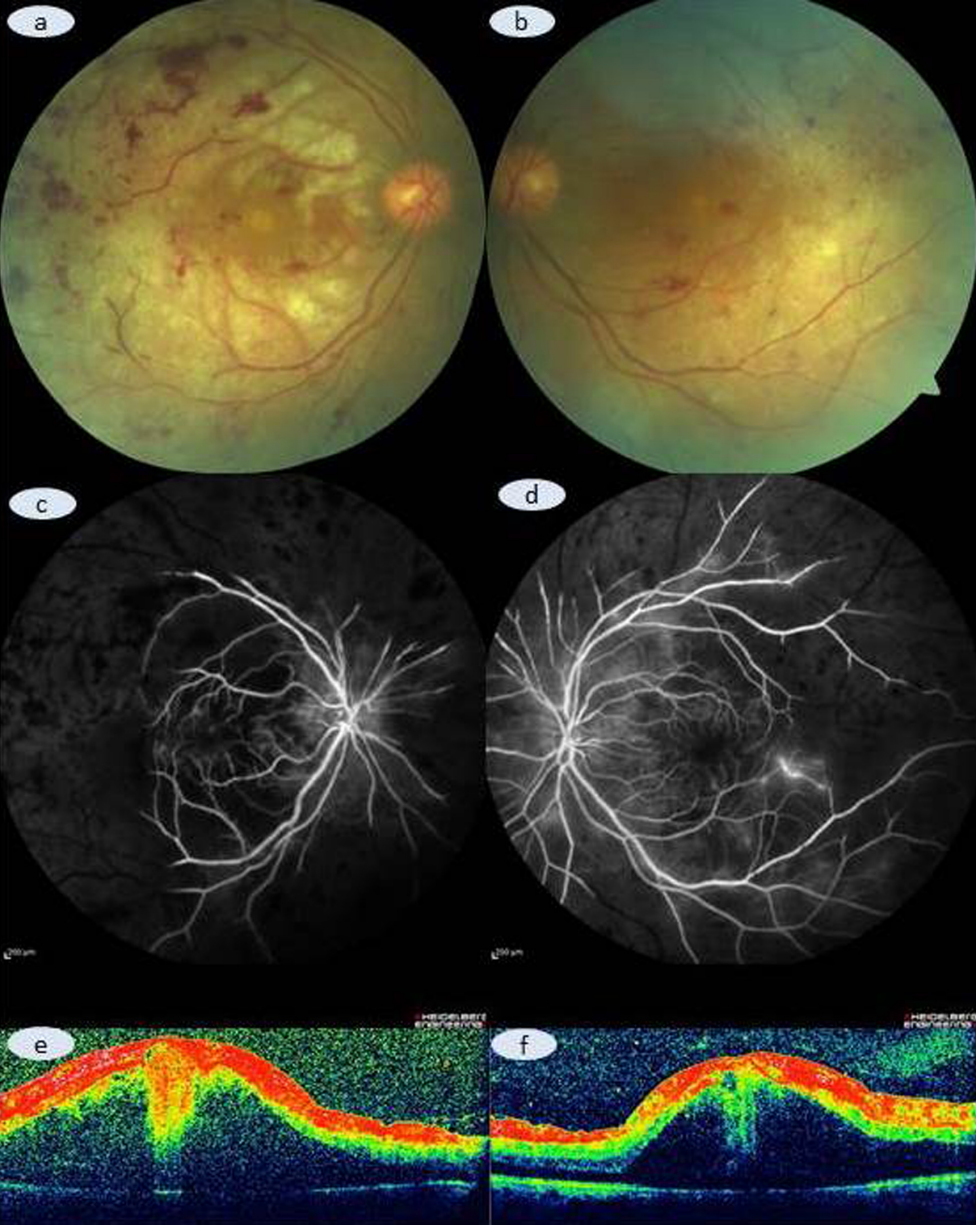
Color fundus picture depicting the whitish appearance of superficial retina with some occluded distal arterioles and some retinal hemorrhages (a: right eye; b: left eye). Venous phase of fluorescein angiogram demonstrating the distal, almost totally occluded arterioles and related capillary hypoperfusion (c: right eye; d: left eye). Optical coherence tomograhy disclosing superficial hyperreflective dots to ganglion layer infarct and severe macular edema (e: right eye; f: left eye).

On our examination, her vision was counting fingers from 1 meter in OD and 2 meters in OS. Slit-lamp examination was unremarkable OU. Intraocular pressure was within normal limits bilaterally. Dilated fundus exam disclosed preretinal hemorrhages, scattered cotton-wool spots and widespread yellowish discoloring of the retina (Figure 1a,b (Fig. 1)). Fluorescein angiogram showed extensive arteriolar closure 360° OU (Figure 1c,d (Fig. 1)). Optical coherence tomography exhibited severe serous macular retinal detachment in both eyes (Figure 1e,f (Fig. 1)). Recent blood investigations revealed a mild leukocytosis (total white cell count 14.6 10^3^/µl with an 80% neutrophilia) [normal, 4–10.3 10^3^/µl]) and mild anemia (hemoglobin: 9.0 g/dL [normal, 12–16 g/dL]). C-reactive protein was 52.2 mg/ml. Serum urea and creatinine levels were 17.4 and 0.38 mg/dL, respectively (normal limits of serum urea and creatinine were 6–20 and 0.51–0.95 mg/dL, respectively). Peripheral blood smear examination showed normochromic normocytic erythrocytes, anisocytosis and neutrophilia. Platelet count was normal. 

**Figure 2:**
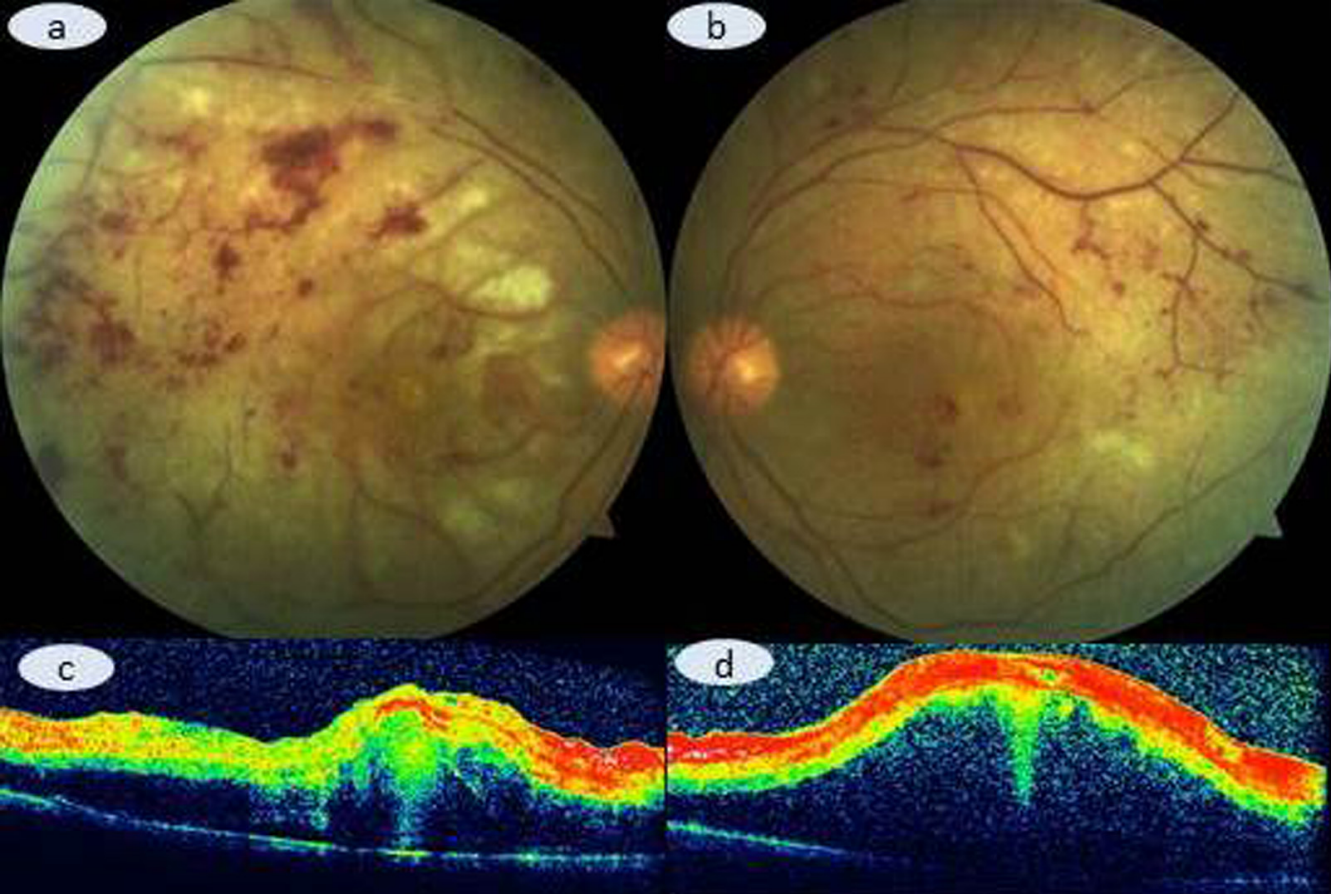
Color fundus picture depicting the slightly increased retinal hemorrhages and continuing retinal whitining (a: right eye; b: left eye). No OCT change is present in the right (c) and left eye (d).

After evaluating the overall clinical situation, brentuximab therapy was discontinued and a diagnosis of Purtscher-like retinopathy in association with brentuximab treatment was assumed. She was hospitalized and received 1 gram of IV methylprednisolone for 3 days. As the visual acuity deteriorated despite the pulse therapy (Figure 2 (Fig. 2)), bilateral simultaneous intravitreal 2 mg aflibecept injection was given after reviewing the therapeutic options with the patient and the family. In order to combat with the systemic disease progression, cyclophosphamide, doxorubicin, vincristine, etoposide, prednisolone (CHOEP) regimen was commenced. One month after the diagnosis of bilateral Purtscher-like retinopathy, her vision was no light perception OU. Her general condition deteriorated and she was transferred to the intensive care unit. She unfortunately passed away almost six weeks after our diagnosis due to respiratory failure.

## Discussion

CD-30 is highly expressed on Reed-Sternberg cells that are the key finding of the Hodgkin’s disease and Brentuximab vedotin causes apoptosis of the CD-30 expressing tumor cells by preventing cell cycle progression of the G_2_ to M phase through the distruption of cytosolic microtuble network. Brentuximab vedotin has some known side effects including peripheral sensory neuropathy and neutropenia that most often can be managed with only dose reduction [4], [5]. No ocular complication was previously reported. 

Many antineoplastic agents can produce various vessel changes ranging from simple phlebitis to even lethal widespread microangiopathy [3]. Purtscher-like retinopathy is most often reported by administration of gemcitabine [6], [7]. Sheyman et al. [6] described a 64-year-old woman who was on chemotherapy regimen including the gemcitabine for the treatment of metastatic cholangiocarcinoma and she developed Purtscher-like retinopathy and nephropathy. Kovach et al. [7] reported a 18-year-old woman who completed 5 cycles of combination treatment with gemcitabine and docataxel for the treatment of leiomyosarcoma and the clinical picture improved after the cessation of only gemcitabine. As the mechanism of Purtscher-like retinopathy, the direct endothelial damage was suggested [8].

## Conclusion

We believe that brentuximab treatment might be linked to occurence of bilateral Purtscher-like retinopathy in the present case with Hodgkin’s lymphoma relapse and thereby we share our experience with the communities of ophthalmology and haematology. 

## Notes

### Competing interests

The authors declare that they have no competing interests.

## References

[1] Agrawal A, McKibbin M (2007). Purtscher’s retinopathy: epidemiology, clinical features and outcome. Br J Ophthalmol.

[2] Miguel AI, Henriques F, Azevedo LF, Loureiro AJ, Maberley DA (2013). Systematic review of Purtscher’s and Purtscher-like retinopathies. Eye (Lond).

[3] Shahab N, Haider S, Doll DC (2006). Vascular toxicity of antineoplastic agents. Semin Oncol.

[4] Scott LJ (2017). Brentuximab Vedotin: A Review in CD30-Positive Hodgkin Lymphoma. Drugs.

[5] Brentuximab vedotin (sgn35) drug description. ADC Review.

[6] Sheyman AT, Wald KJ, Pahk PJ, Freund KB (2014). Gemcitabine associated retinopathy and nephropathy. Retin Cases Brief Rep.

[7] Kovach JL (2016). Gemcitabine-induced retinopathy. Retin Cases Brief Rep.

[8] Tran TH, Desauw C, Rose C (2009). Gemcitabine-induced retinopathy in a diabetic patient. Acta Ophthalmol.

